# Toxicity of Antioxidant Supplements in Patients with Male Factor Infertility: A Systematic Review and Meta-Analysis of Randomized Controlled Trials

**DOI:** 10.3390/antiox11010089

**Published:** 2021-12-30

**Authors:** Massimiliano Creta, Davide Arcaniolo, Giuseppe Celentano, Luigi Napolitano, Roberto La Rocca, Marco Capece, Gianluigi Califano, Francesco Mangiapia, Lorenzo Spirito, Felice Crocetto, Ciro Imbimbo, Nicola Longo, Marco De Sio, Ferdinando Fusco

**Affiliations:** 1 Department of Neurosciences, Science of Reproduction and Odontostomatology, University of Naples Federico II, 80131 Naples, Italy; max.creta@gmail.com (M.C.); dr.giuseppecelentano@gmail.com (G.C.); robertolarocca87@gmail.com (R.L.R.); drmarcocapece@gmail.com (M.C.); gianl.califano2@gmail.com (G.C.); mangiapiaf@gmail.com (F.M.); lorenzospirito@msn.com (L.S.); ciro.imbimbo@unina.it (C.I.); nicolalongo20@yahoo.it (N.L.); 2 Department of Woman, Child and General and Specialized Surgery, Urology Unit, University of Campania ‘Luigi Vanvitelli’, 80131 Naples, Italy; davide.arcaniolo@gmail.com (D.A.); marco.desio@unicampania.it (M.D.S.); ferdinando-fusco@libero.it (F.F.); 3 Department of General and Specialized Surgeries, Renal Transplantation, Nephrology, Care and Pain Management, University of Federico II, 80100 Naples, Italy; felice.crocetto@unina.it

**Keywords:** adverse events, antioxidants, male infertility, meta-analysis

## Abstract

Treating oxidative stress through antioxidant therapy has been considered an appealing strategy in the management of male infertility. However, evidence regarding the toxicity of antioxidant therapy is controversial. We summarized the available clinical evidence on the toxicity associated with the use of antioxidants in infertile males. A systematic review was performed in March 2021. We included randomized controlled trials evaluating the incidence of adverse events in male patients with infertility receiving antioxidant therapy. Thirteen studies involving 1999 male patients were identified. Antioxidant supplementation in patients with male factor infertility was associated with a statistically significantly increased risk of nausea (Odds Ratio: 2.16, 95% Confidence Interval: 1.05–4.43, *p* = 0.036), headache (Odds Ratio: 3.05, 95% Confidence Interval: 1.59– 5.85 *p* = 0.001), and dyspepsia (Odds Ratio: 4.12, 95% Confidence Interval: 1.43–11.85, *p* = 0.009) compared to a placebo. Treatment discontinuation due to adverse events was not significantly higher in patients taking antioxidants compared to a placebo (Odds Ratio: 2.29, 95% Confidence Interval: 0.76–6.88, *p* = 0.139). When antioxidant supplementation is considered, a more accurate risk/benefit analysis is warranted.

## 1. Introduction

Infertility, defined as the inability of a couple to achieve a pregnancy after one year of regular unprotected intercourse, affects 10 to 15% of couples [1]. It has been reported that a male factor is present in about 20–70% of infertile couples [1,2,3]. Oxidative stress (OS), defined as an imbalance in the levels of reactive oxygen species (ROS) and antioxidants, has been reported as one of the main causes of male infertility [4]. Spermatozoa are highly sensitive to OS [4]. Indeed, these cells are incapable of repairing damage caused by OS because they suffer from a lack of essential cytoplasmic enzymes [4]. Moreover, OS can also interfere with the hypothalamic axis and disrupt the secretion of sex hormones [4,5]. In recent years, treating OS through either ROS reduction or antioxidant therapy has been considered an appealing strategy in the management of male infertility; in everyday clinical practice, physicians usually prescribe antioxidant supplements to treat cases of male infertility [6,7]. However, high doses of antioxidants can produce adverse effects resulting from the imbalance in the physiological redox status through a phenomenon called “reductive stress” or the “antioxidant paradox” [4]. Therefore, some authors suggest caution in considering the prescription of antioxidants and individualizing treatment based on patients’ redox status [7]. Herein, we aimed to summarize the available clinical findings related to toxicity associated with the use of antioxidants in infertile males.

## 2. Materials and Methods

The present analysis was conducted and reported according to the general guidelines recommended by the Primary Reporting Items for Systematic Reviews and Meta-Analyses (PRISMA) statement [8]. This protocol was registered in PROSPERO (ID 292202).

### 2.1. Literature Search

The search was performed in the Medline (US National Library of Medicine, Bethesda, MD, USA), Scopus (Elsevier, Amsterdam, The Netherlands), and Web of Science Core Collection (Thomson Reuters, Toronto, ON, Canada) databases up to March 2021. The following terms were combined to capture relevant publications: (“antioxidants” OR “oxidative stress” OR “reactive oxygen species”) AND (“infertility” OR “fertility”). Reference lists in relevant articles and reviews were also screened for additional studies.

### 2.2. Selection Criteria and Data Collection

Two authors (L.N. and F.F.) reviewed the records separately and individually to select relevant publications, with any discrepancies resolved by a third author (M.C.). To assess eligibility for the systematic review, PICOS (participants, intervention, comparisons, outcomes, and study type) criteria were used [9]. PICOS criteria were set as follows: (P)articipants—patients with male factor infertility; (I)ntervention—antioxidant supplementation; (C)omparator—patients not receiving antioxidants; (O)utcome—adverse events; (S)tudy types—randomized controlled trials (RCT). The following data were extracted: first author, study design, sample size, patients’ age, infertility characteristics, the antioxidant evaluated, antioxidant dosage, control arm, treatment duration, significant benefits in the experimental arm, adverse events, and discontinuations due to adverse events. The quality of included studies was assessed using the Jadad score [10].

### 2.3. Statistical Analysis

The meta-analysis was performed using ProMeta 3 software when two or more studies reported the same outcome under the same definition. The effect size (ES) was estimated using an odds ratio (OR) reported with its 95% confidence interval (CI). Heterogeneity among studies was evaluated using the I2 statistics. A *p* < 0.05 was considered statistically significant. To calculate the pooled effect, a random effect model was applied. Egger’s linear regression test and Begg and Mazumdar’s rank correlation test were also used to evaluate the publication bias of studies included in the meta-analysis.

## 3. Results

**Figure 1:**
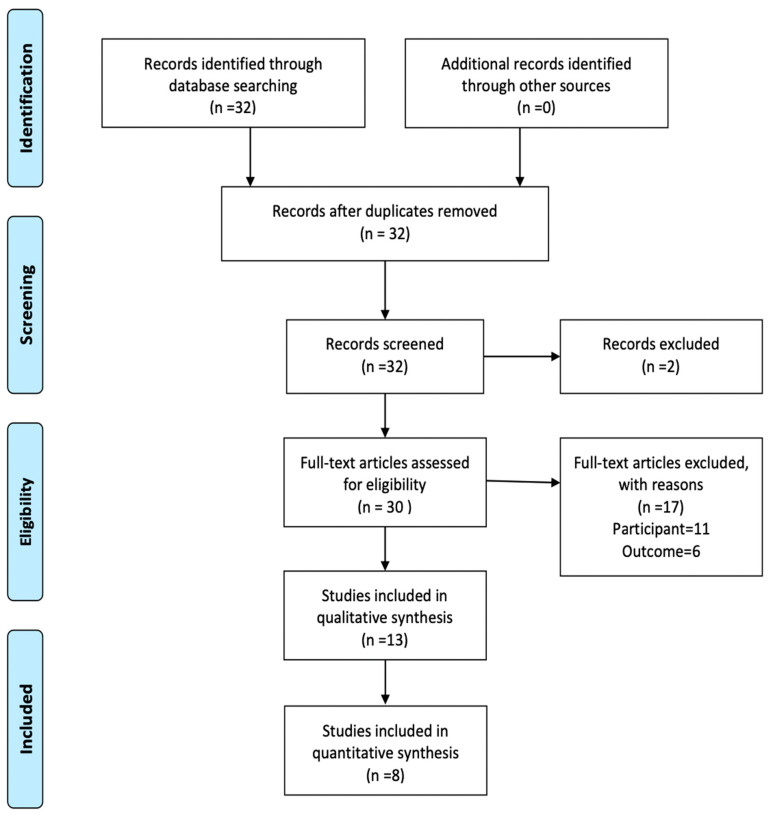
PRISMA study flow.

The search strategy revealed a total of 32 results. The screening of the titles and abstracts determined 30 papers eligible for inclusion. Further assessment of eligibility, based on the study of the full-text papers, led to the exclusion of 17 papers. Finally, 13 RCTs involving 1999 patients were included in the final analysis (Figure 1) [11,12,13,14,15,16,17,18,19,20,21,22,23].

The study characteristics, patients’ demographics, and treatment features are summarized in Table 1.

Eleven RCTs were double-blind and two were triple-blind. The study quality was considered high (Jadad score ≥ 3) in all cases. The mean age of patients ranged from 23.3 to 34.7 years. The occurrence of adverse events in the experimental arm was reported in 8 (57.1%) studies. Table 2 describes the characteristics of adverse events reported by the included studies in the active treatment and control arms.

**Figure 2:**
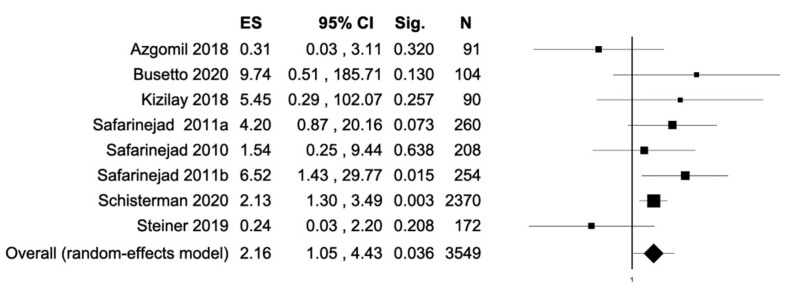
Forest plot showing the OR for nausea. ES, effect size; CI, confidence interval. (I2 = 34.81, *p* = 0.150).

**Figure 3:**
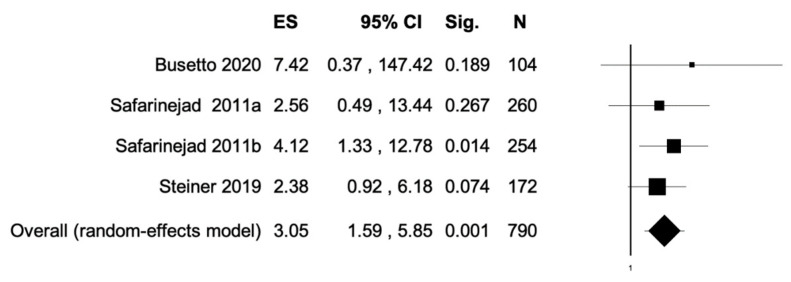
Forest plot showing the OR for headache. ES, effect size; CI, confidence interval. (I2 = 0.00, *p* = 0.823).

**Figure 4:**
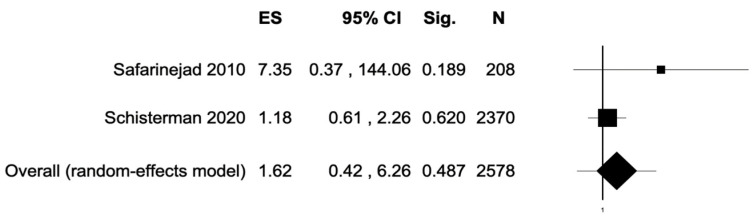
Forest plot showing the OR for pruritus. ES, effect size; CI, confidence interval. (I2 = 27.82, *p* = 0.239).

**Figure 5:**
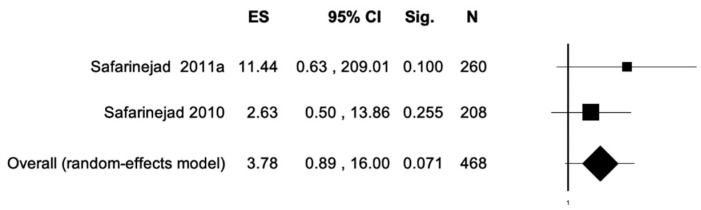
Forest plot showing the OR for diarrhea. ES, effect size; CI, confidence interval. (I2 = 0.00, *p* = 0.389).

**Figure 6:**
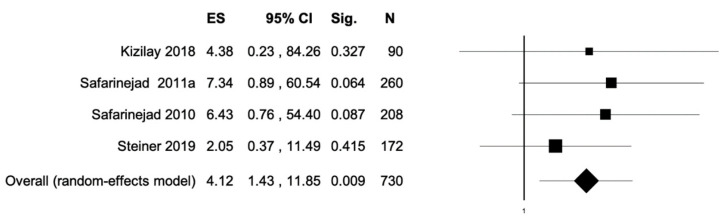
Forest plot showing the OR for dyspepsia. ES, effect size; CI, confidence interval. (I2 = 0.00, *p* = 0.780).

**Figure 7:**
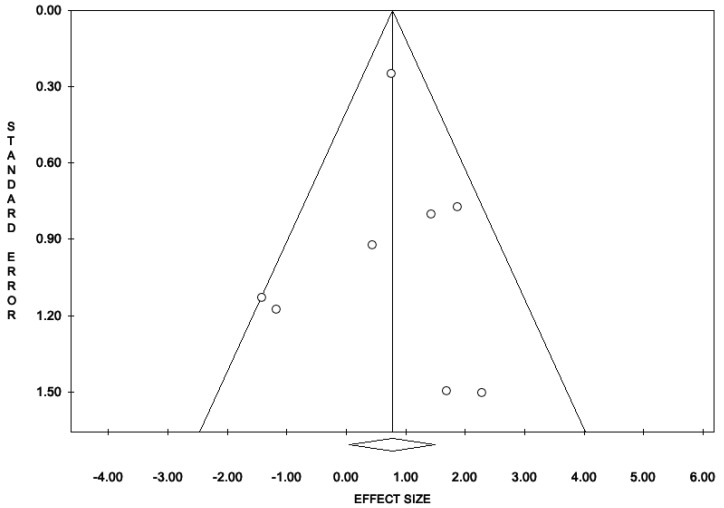
Funnel plots of the meta-analysis evaluating the OR for nausea. Egger’s linear regression (t = −0.10, *p* = 0.921) and Begg and Mazumdar rank correlation test (z = −0.25, *p* = 0.805).

**Figure 8:**
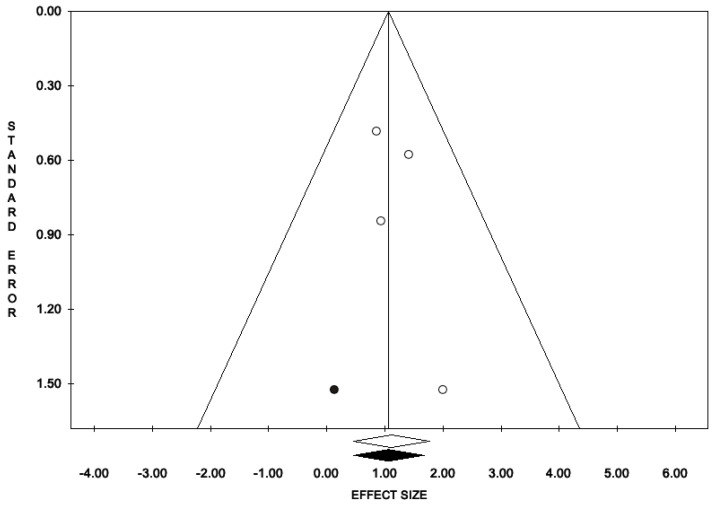
Funnel plots of the meta-analysis evaluating the OR for headache. Egger’s linear regression (t = 1, *p* = 0.42) and Begg and Mazumdar rank correlation test (z = 0.68, *p* = 0.497).

**Figure 9:**
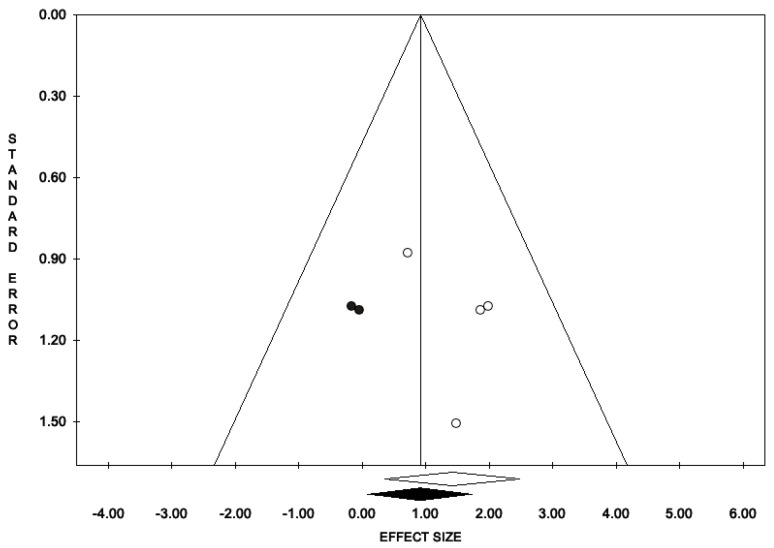
Funnel plots of the meta-analysis evaluating the OR for dyspepsia. Egger’s linear regression (t = 0.85, *p* = 0.486) and Begg and Mazumdar rank correlation test (z = 0.00, *p* = 1.00).

**Figure 10:**
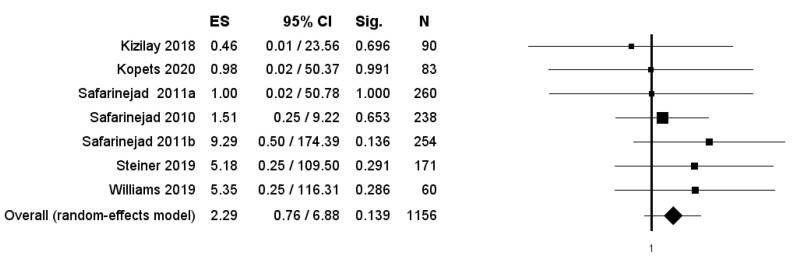
Forest plot showing the OR for treatment discontinuation due to adverse events. ES: effect size; CI, confidence interval. (I2 = 0.00, *p* = 0.853).

The percentage of patients reporting adverse events ranged from 0.8% to 60%. The most commonly reported adverse events were nausea, headache, pruritus, diarrhea, and dyspepsia. Treatment discontinuation due to adverse events in the experimental arm was described in 10 (0.5%) patients. Pooled data from studies reporting nausea, headache, pruritus, diarrhea, and dyspepsia as adverse events are reported in Figure 2, Figure 3, Figure 4, Figure 5 and Figure 6. The meta-analysis demonstrated a statistically significantly higher OR for nausea, headache, and dyspepsia in patients receiving antioxidant therapy. Bias evaluation is reported in (Figure 7, Figure 8 and Figure 9). The OR of discontinuation due to adverse events was not statistically significantly higher in patients treated with antioxidants compared to a placebo (Figure 10).

## 4. Discussion

Sperm damage induced by ROS is involved in 30–80% of cases of male infertility [24]. Although ROS have a crucial role in allowing sperm capacitation and acrosomal reaction, sperm cells are highly sensitive to OS as they are not able to defend themselves. Therefore, any imbalance in ROS production can lead to sperm damage and male infertility [25]. Antioxidants are used on a very large scale to preserve optimal health. The putative effectiveness of antioxidants in improving semen parameters reducing OS seems to demonstrate the causative nature of this association. Data from clinical trials are controversial, ranging from increasing semen parameters to no clinical improvement or even harmful effects [26,27,28,29,30]. This inconsistency in clinical trials is probably due to small sample sizes, the lack of a control group, and non-standardized treatment regimens in terms of duration and dose. Finding the right dosage of antioxidants represents a crucial point, as a low dose could lead to ineffective treatment, while an excess of antioxidants could result in significant adverse events and even promote reductive stress, which is as detrimental as OS for male fertility [7,31]. In addition, only a few studies used pregnancy rates and live birth rates as primary outcomes. Results from a Cochrane review showed higher live birth and pregnancy rates in patients treated with antioxidants compared to a placebo or no treatment; even when studies with a high risk of bias were excluded from the analysis, the resulting difference was not statistically significant [32]. Despite these conclusions, the lack of high-quality evidence still represents a major issue for clinicians. In fact, most systematic reviews and meta-analyses on the topic showed an overall low quality of included studies; therefore, no clear recommendation for antioxidant therapy can be drawn [6,33,34]. Nevertheless, in a recent survey, more than 85% of clinicians worldwide stated that they recommend antioxidant therapy in infertile males [35]. Of note, recent evidence suggests that antioxidant supplements may be harmful and cause unwanted consequences to health [36,37,38,39,40]. Currently, however, we lack detailed knowledge of the adverse events profile of antioxidants used in various clinical settings. To the best of our knowledge, we performed the first systematic review and meta-analysis evaluating the adverse event profile of antioxidants used in infertile males. We found a significantly higher risk of nausea, headache, and dyspepsia in patients undergoing antioxidant therapy compared to a placebo or no treatment. These results are in line with findings from an updated Cochrane review by Smits et al. that showed an increased risk of mild gastrointestinal events in patients taking antioxidants [32]. Clinical experience strongly suggests that these adverse events may occur with almost any medication, and the exact pathophysiology of these adverse events in patients taking antioxidants remains poorly understood. However, some authors have hypothesized that ROS are involved in many physiological conditions, including the physiology of the gastrointestinal tract, and the excess of exogenous antioxidants may be involved in the so-called “reductive stress” that may be responsible for the detrimental consequences of antioxidants [41,42]. Accordingly, there is evidence that the beneficial effects of antioxidants depend on their concentration and that health benefits are mainly observed when they are consumed within their natural source rather than in supplements, where the dosage is significantly higher. This is probably due to the synergistic effect of the relatively low concentration of nutrients with other compounds detectable in food, which are not present in available supplements [36]. So, contrary to what has always been thought, the higher the concentration of antioxidants the higher the risk of detrimental effects. Despite this evidence, only a small percentage (about 35%) of clinicians report the use of routine OS tests in their clinical practice to evaluate the oxidation-reduction balance before starting treatment [35]. Interestingly, the OR for drug discontinuation due to adverse events was not statistically significantly higher in patients taking antioxidants compared to controls. The limits of the present review reflect the limits of the studies included. These mainly include low numbers of patients and heterogeneity in terms of baseline clinical features, the type and dosage of antioxidants, and the duration of treatment. Of note, the studies lacked standardized protocols for assessing and reporting complications. Taking this into account, the potentially detrimental effect of antioxidant therapy should be considered before starting treatment in order to avoid systemic adverse events. Careful patient selection for antioxidant therapy represents a challenging issue. Several lines of evidence support the inclusion of tests for the assessment of seminal OS to the male infertility workup algorithms and several tests have been introduced [43,44]. Traditionally, OS evaluation involved ROS level measurements in seminal plasma. However, a new methodology based on an electrochemical analysis of the oxidation-reduction potential—the MiOXSYS system—has recently been developed [45]. Unfortunately, these tests are poorly adopted in everyday clinical practice, and antioxidants are prescribed empirically. The potential benefits of such evaluations include the accurate selection of patients who could benefit from antioxidant therapies, the customization of dosages, the monitoring of benefits, and the avoidance of adverse events when not indicated. Moreover, all potential determinants of OS should be investigated and corrected, when possible. Finally, the side effects profile of antioxidants used for other conditions should be investigated [45].

## 5. Conclusions

Antioxidant supplementation in patients with male factor infertility is associated with a statistically significantly increased risk of nausea, headache, and dyspepsia compared to a placebo or no treatment. However, treatment discontinuation due to adverse events is not significantly higher, thus suggesting their mild nature. When antioxidant supplementation therapy is considered for infertile males, accurate counseling about the risk/benefit ratio is warranted.

## Figures and Tables

**Table 1:** Study characteristics, patients’ demographics, and treatment features.

Study	Design	JS	Treatment Arm (*n*)	Age Mean (SD)	Infertility Characteristics	Drug, Dosage/Day	Control Arm (*n*)	Treatment Duration (Weeks)	Significant Findings
Safarinejad [11]	DB-RCT	4	130	28.6 (5.4)	Idiopathic OAT	Saffron, 60 mg	Placebo (130)	26	-
Safarinejad [12]	DB-RCT	5	119	32.0 (9.0)	Primary infertility and idiopathic OAT	Omega-3 (EPA or DHA), 1.84 g	Placebo (119)	32	↑ SM ↑ SMo
				32.0 (10.0)					
Safarinejad [13]	DB-RCT	5	127	32.1 (4.3)	Infertile men	Pentoxifylline, 400 mg	Placebo (127)	24	↑ SC ↑ SM ↑ SMo
Haghighian [14]	TB-RCT	5	23	32.9 (5.3)	SMo < 50%, FM < 25%	ALA, 600 mg	Placebo (21)	12	↑ SC ↑ SMo ↑ TSC
				34.1 (4.7)					
Alizadeh [15]	DB-RCT	5	30	30.5 (4.0)	Idiopathic OAT	Curcumin, 80 mg	Placebo (30)	10	↑ SC ↑ SM ↑ TSC
				30.0 (3.9)					
Azgomi [16]	TB-RCT	5	46	32.5 (5.5)	Inability to conceive after 1 year, SC < 20 million/mL, SM < 30%, SMo < 50%	Withania somnifer, 5 mg	Pentoxifyllin, 80 mg (45)	12	↑ EV ↑ SC ↑ SM ↑ SMo
				34.7 (5.6)					
Steiner [17]	DB-RCT	3	85	34.0 (n/a)	Men with SC < 15 million/mL, SMo < 40%, SM < 4%, or DNA fragmentation > 25%	Supplement × 1 Vitamin C, 500 mg Vitamin E, 400 mg Selenium, 0.2 mg L-carnitine, 1000 mg Zinc, 20 mg Folic acid, 1000 mg Lycopene, 10 mg Vitamin D, 2000 IU	Placebo (86)	24	-
				34.0 (n/a)					
Kizilay [18]	DB-RCT	3	62	32.8 (3.1)	Infertile patients after varicocelectomy	Supplement × 2 L-carnitine fumarate, 1 g Acetyl-L-carnitine HCl, 0.5 g Fructose, 1 g Citric acid, 50 mg Folic acid, 200 mcg Vitamin C, 90 mg Zinc, 10 mg Selenium, 50 mcg Coenzyme Q10, 20 mg Vitamine B12, 1.5 mcg	No treatment (28)	24	↑ SC ↑ SM ↑ SMo ↑ TSC
				32.1 (2.4)					
Williams [19]	DB-RCT	5	30	23.3 (2.9)	Healthy men	Lactolycopen, 14 mg	Placebo (30)	12	↑ SM ↑ SMo
				23.3 (2.5)					
Schisterman [20]	DB-RCT	5	1185	32.5 (5.7)	Male partners of infertile couple	Folic acid, 5 mg Elemental zinc, 30 mg	Placebo (1185)	24	-
				32.7 (6.0)					
Busetto [21]	DB-RCT	4	52	32.5 (n/a)	Oligo and/or as- theno- and/or teratozoospermia with or without varicocele	Supplement × 2 L-carnitine fumarate, 1 g Acetyl-L-carnitine HCl, 0.5 g Fructose, 1 g Citric acid, 50 mg Folic acid, 200 mcg Vitamin C, 90 mg Zinc, 10 mg Selenium, 50 mcg Coenzyme Q10, 20 mg Vitamine B12, 1.5 mcg	Placebo (52)	24	↑ SM ↑ TSC
Kopets [22]	DB-RCT	5	42	32.5 (6.1)	Oligo and/or astheno- and/or teratozoospermia	Supplement × 3 L-carnitine/Acetyl-L-carnitine, 1990 mg L-arginine, 250 mg Glutathione, 100 mg Co-enzyme Q10, 40 mg Zinc, 7.5 mg Vitamin B12, 2 mcg Selenium, 50 mcg	Placebo (41)	24	↑ PM ↑ SC ↑ SMo
				32.7 (5.2)					
Eslamian [23]	DB-RCT	5	45	32.7 (4.4)	SMo < 40% PM < 32%	DHA, 465 mg + vitamin E, 600 IU	Placebo (135)	12	↑ SC ↑ SM ↑ SMo ↑ TSC
				32.7 (4.4)					

ALA, alpha-lipoic acid; DHA, docosahexaenoic acids; DB-RCT, double-blind-randomized controlled trial; EPA, eicosapentaenoic; EV, ejaculate volume; IU, international unit; JS, Jadad score; n/a, not available; PM, progressive motility; SC, sperm concetration; SD, standard deviation; SM, sperm morphology; SMo, sperm motility; TB-RCT, triple-blind-randomized controlled trial; TSC, total sperm count; ↑, increase.

**Table 2:** Adverse events reported by the included studies in the experimental and control arms.

Study	Adverse Events Type, *n* (%)		Discontinuation Due to Adverse Events Type, *n* (%)	
	Experimental Arm	Control Arm	Experimental Arm	Control Arm
Safarinejad [11]	Nausea, 8 (6.4) Vomiting, 8 (6.4) Dyspepsia, 7 (5.6) Headache, 5 (4.0) Diarrhea, 5 (4.0) Tremor, 2 (1.6) Dizziness, 2 (1.6) Vertigo, 2 (1.6)	Nausea, 2 (1.6) Headache, 2 (1.6) Vomiting, 1 (0.8) Dyspepsia, 1 (0.8) Vertigo, 1 (0.8)	0	0
Safarinejad [12]	Foul breath/bad taste, 8 (7.1) Heartburn/reflux, 6 (5.3) Soft stool or diarrhea, 5 (4.4) Nausea, 3 (2.6) Constipation, 3 (2.6) Pruritis, 3 (2.6) Loss of body weight, 1 (0.9) Burping, 1 (0.9) Feeling tired after starting medication, 1 (0.9)	Foul breath/bad taste, 1 (0.9) Heartburn/reflux, 1 (0.9) Soft stool or diarrhea, 2 (1.8) Nausea, 2 (1.8) Constipation, 1 (0.9)	Rectorrhagia, pruritus, diarrhea, 3 (2.5)	n/a, 2 (1.7)
Safarinejad [13]	Decreased platelet count, 81 (62.3) Decreased leukocyte, 78 (60.0) Decreased red blood cell, 72 (55.4) Decreased appetite, 17 (13.1) Increased appetite, 17 (13.1) Headache, 15 (11.5) Nausea, 12 (9.2) Sedaction, 10 (7.7) Hypomania, 10 (7.7)	Headache, 4 (3.1) Nausea, 2 (1.5) Decreased appetite, 1 (0.7) Increased appetite, 1 (0.7)	n/a, 4 (3.1)	0
Haghighian [14]	0	0	n/a	n/a
Alizadeh [15]	0	0	n/a	n/a
Azgomi [16]	Nausea and epigastric pain, 1 (2.7)	Nausea and epigastric pain, 3 (6.6)	n/a	n/a
Steiner [17]	Headache, 15 (17.6) Upper respiratory infection, 4 (4.7) Dyspepsia, 4 (4.7) Nasopharyngitis, 4 (4.7) Nausea, 1 (1.2)	Headache, 7 (8.1) Nasopharyngitis, 7 (8.1) Abdominal pain, 4 (4.7) Dyspepsia, 2 (2.3) Nausea, 4 (4.7) Upper respiratory infection, 4 (4.7)	n/a, 2 (2.3)	0
Kizilay [18]	Nausea, 5 (8.1) Gastroesophageal reflux, 4 (6.4)	0	0	0
Williams [19]	0	0	Sleeping difficulty, 2 (6.6)	0
Schisterman [20]	Abdominal discomfort, 66 (5.6) Pyrexia, 66 (5.6) Oropharyngeal pain, 57 (4.8) Nausea, 50 (4.2) Vomiting, 32 (2.7) Nasopharyngitis, 32 (2.7) Erythema, 23 (1.9) Influenza, 21 (1.8) Pruritus, 20 (1.7) Rash, 21 (1.8)	Pyrexia, 62 (5.2) Oropharyngeal pain, 60 (5.1) Nasopharyngitis, 40 (3.3) Abdominal discomfort, 40 (3.3) Nausea, 24 (2.0) Vomiting, 17 (1.4) Rash, 12 (1.0) Influenza, 11 (0.9) Erythema, 8 (0.7)	n/a	n/a
Busetto [21]	Nausea, 4 (7.7) Vertigo or headache, 3 (5.8)	0	n/a	n/a
Kopets [22]	0	0	0	0
Eslamian [23]	0	0	n/a	n/a
